# Combination treatment with ethyl pyruvate and IGF-I exerts neuroprotective effects against brain injury in a rat model of neonatal hypoxic-ischemic encephalopathy

**DOI:** 10.3892/ijmm.2015.2219

**Published:** 2015-05-22

**Authors:** ZHIHUI RONG, RUI PAN, LIWEN CHANG, WEIHUA LEE

**Affiliations:** 1 Department of Pediatrics, Tongji Hospital, Tongji Medical College, Huazhong University of Science and Technology, Wuhan, Hubei 430030, P.R. China; 2 Department of Pediatrics, Indiana University School of Medicine, Indianapolis, IN 46202, USA

**Keywords:** hypoxic-ischemic encephalopathy, oxygen glucose deprivation, ethyl pyruvate, insulin-like growth factor-I, antioxidants

## Abstract

Neonatal hypoxic-ischemic (HI) brain injury causes severe brain damage in newborns. Following HI injury, rapidly accumulating oxidants injure neurons and interrupt ongoing developmental processes. The antioxidant, sodium pyruvate, has been shown to reduce neuronal injury in neonatal rats under conditions of oxygen glucose deprivation (OGD) and HI injury. In this study, we evaluated the effects of ethyl pyruvate (EP) and insulin-like growth factor-I (IGF-I) alone or in combination in a similar setting. For this purpose, we used an *in vitro* model involving primary neonatal rat cortical neurons subjected to OGD for 2.5 h and an *in vivo* model involving unilateral carotid ligation in rats on post-natal day 7 with exposure to 8% hypoxia for 2.5 h. The cultured neurons were examined by lactate dehydrogenase (LDH) and cell viability assays. For the *in vivo* experiments, behavioral development was evaluated by the foot fault test at 4 weeks of recovery. 2,3,5-Triphenyltetrazolium chloride monohydrate and cresyl violet staining were used to evaluate HI injury. The injured neurons were Fluoro-Jade B-labeled, new neuroprecursors were double labeled with bromodeoxyuridine (BrdU) and doublecortin, new mature neurons were BrdU-labeled and neuronal nuclei were labeled by immunofluorescence. Under conditions of OGD, the LDH levels increased and neuronal viability decreased. Treatment with 0.5 mM EP or 25 ng/ml IGF-I protected the neurons (P<0.05), exerting additive effects. Similarly, either the early administration of EP or delayed treatment with IGF-I protected the neonatal rat brains against HI injury and improved neurological performance and these effects were also additive. This effect may be the result of reduced neuronal injury, and enhanced neurogenesis and maturation. On the whole, our findings demonstrate that the combination of the early administration of EP with delayed treatment with IGF-I exerts neuroprotective effects against HI injury in neonatal rat brains.

## Introduction

Immature brains are known to be exceedingly sensitive to oxidative stress, due to high levels of unsaturated fatty acids, a high rate of oxygen consumption, low concentrations of antioxidants, a high content of metals catalyzing free radical formation and a large proportion of sensitive immature cells (1–3). This sensitivity undoubtedly contributes to the severe neuronal damage observed following hypoxic-ischemic encephalopathy (HIE) in newborns (3). HIE occurs in 0.1–0.2% of term or near-term infants, among whom approximately 20% die and up to 40% of the survivors often suffer devastating disabilities, such as cerebral palsy, mental retardation and epilepsy (4–7). Thus, the reduction of damaging oxidants in neonatal brains following hypoxic-ischemic (HI) injury is one of the main therapeutic goals for the effective treatment of HIE.

Compared with adult patients, infants have a poorer prognosis as HI injury not only leads to injury to neuronal cells, but it also disrupts ongoing brain development. Insulin-like growth factor-I (IGF-I) is an essential trophic factor for neuronal proliferation and differentiation (8,9). Its potential neuroprotective effects against HI injury in immature brains have been evaluated in fetal sheep (10) and neonatal rat models (11). In the rat model, although the exogenous administration of IGF-I was shown to exert neuroprotective effects and to improve long-term neurological function (11), only a 40% reduction in brain injury was achieved at 3 days of recovery when IGF-I was administered by intraventricular injection immediately following HI injury (11). On the other hand, the delayed subcutaneous administration of IGF-I (24 and 48 h post-injury) has been shown to significantly increase the volume of surviving brain tissue and to improve behavioral development at 2 months of age (19). These time-dependent neuroprotective effects may be attributed to the suppression of IGF-I activity due to increased levels of oxidative stress in the acute phase of injury. Therefore, we hypothesized that the reduction of the levels of oxidative stress may enhance the neuroprotective effects of IGF-I.

Sodium pyruvate (SP) is a substrate of the tricarboxylic acid cycle and an extracellular antioxidant (12,13). In a previous study, we found that SP improved the survival of primary cortical neurons under conditions of oxygen glucose deprivation (OGD) and, when administered 30 min after HI injury, it reduced HI injury to neonatal rat brains and improved long-term behavioral recovery (14). Compared with SP, ethyl pyruvate (EP) is a more stable lipophilic ester derivative of pyruvate and has been proven to reduce HI injury to neonatal brains through anti-cell death and anti-inflammatory mechanisms (15). As EP has a longer half-life and fewer side-effects, we hypothesized that the early administration of EP may improve the protective effects of IGF-I against HI in immature brains. In this study, we examined this hypothesis *in vitro* using a model of ODG, as well as *in vivo* using a model of neonatal rat HI injury.

## Materials and methods

### Primary cortical neuron culture

The animal experiments were approved by the Ethics Committee of Indiana University School of Medicine, Indianapolis, IN, USA. The cultured primary neurons were derived from newborn Sprague-Dawley rats (10–12 pups) as previously described (6). Briefly, after removing the meninges, the cortical tissue was minced and maintained in Dulbecco’s modified Eagle’s medium (DMEM) at 4°C. An aliquot of 0.25% trypsin (15050-065; Invitrogen, Carlsbad, CA, USA) and DNase (DN25; Sigma, St. Louis, MO, USA) was added to the tissue and incubated for 15 min at 37°C to produce a single cell suspension. Following centrifugation, the cells were resuspended in neurobasal (NB) medium (10888; Gibco/Life Technologies, Grand Island, NY, USA) supplemented with 2% B-27 (17504-044; Invitrogen), 0.5 mM glutamine (25030-081; Gibco), 100 U/ml penicillin and 100 *µ*g/ml streptomycin. The cells were plated into poly-L-lysine-coated (P1399; Sigma) dishes at 5×10^5^/ml. The culture medium was changed at 24 h and 4 days *in vitro* (DIV) and fibroblast growth factor (FGF; 5 ng/ml final concentration; F0291; Sigma) was added to the culture medium. The cells were ready to use at 6–7 DIV.

### OGD

On day 7, the cultured primary cortical neurons were gently washed with phosphate-buffered saline (PBS) and the medium was then changed to glucose-free DMEM (11965; Gibco) before the cells were placed in a humidified chamber gassed with 95% N_2_/5% CO_2_ at 37°C. The medium of the control cells was changed to DMEM with glucose (11966; Gibco) and the cells were kept in a regular incubator (5% CO_2_ and 21% O_2_, 37°C). After 2.5 h, the cells were removed from the hypoxic chamber to the regular incubator after changing the medium back to NB medium and treated with EP (0.5 mM; E47808; Sigma) and/or IGF-I (25 ng/ml; 100-11; Peprotech, Inc., Rocky Hill, NJ, USA).

### Lactate dehydrogenase (LDH) release and MTT assay

Cell injury was assessed by measuring the amount of LDH released into the culture medium using a cytotoxicity detection kit (G1780; Promega Corp., Madison, WI, USA) according to the manufacturer’s instructions. Briefly, 50 *µ*l of culture medium were mixed with 50 *µ*l substrate followed by incubation in the dark at 37°C for 30 min. Subsequenlty, 50 *µ*l stop solution were added and the absorbance was measured at 490 nm using a microplate reader (M2003; Sigma).

Cell viability was monitored by MTT colorimetric assay (M2003; Sigma). A total of 10 *µ*l MTT was added to 500 *µ*l of cell culture medium followed by incubation at 37°C for 4 h. After discarding the medium, 500 *µ*l dimethyl sulfoxide were added and the absorbance at 570 nm was recorded using a microplate reader (M2003; Sigma).

### Model of neonatal HI injury

Briefly, 7-day-old Sprague-Dawley rat pups (8 per litter, weighing 13–18 g) were anesthetized with a mixture of isoflurane (3–4% for induction and 2% for maintenance) and 30% O_2_/70% N_2_. The left carotid artery of each pup was exposed and ligated with 6-0 surgical silk. After a 2-h recovery period, the pups were placed in 2-litre airtight and watertight glass flasks, submerged in a 37.0°C water bath, and exposed to a humidified mixture of 8% O_2_ and 92% N_2_. After 2.5 h of hypoxia, the pups were then returned to their dams and received the different treatments. The environmental temperature following HI injury was 25°C. EP was administered 30 min after HI injury by intraperitoneal (i.p.) injection (25 mg/kg; E47808; Sigma) and IGF-I was administered 24 h after HI injury by subcutaneous (s.c.) injection (3 mg/kg; 100-11; Peprotech, Inc.). The same volume of normal saline was injected and served as the vehicle. The sham-operated group underwent the same surgical procedure apart from carotid artery ligaton and exposure to hypoxia.

### Foot fault test

Foot-fault tests were performed at 4 weeks of recovery as previously described (14). The rats were placed on an elevated stainless steel wire (diameter, 0.4 cm) grid (1 m above the floor with 3 cm^2^ holes). Each pup was placed on the grid and the number of foot faults was counted out of 50 steps for forelimbs or hindlimbs. A foot fault was defined as when the animal misplaced a forelimb or hindlimb and the paw fell between the grid bars. The examiners were blinded from the study design.

### 2,3,5-Triphenyltetrazolium chloride monohydrate (TTC) staining

TTC staining was performed as previously described (16). At 48 h after HI injury, the rat brains were removed after sacrifice and immediately sectioned coronally into 6 slices (2-mm-thick) in a brain matrix (RBM-4000C Rodent Brain Matrix, Adult Rat, Coronal Sections; ASI Instruments, Inc. Warren, MI, USA). The brain slices were incubated in TTC (T8877; 1%; Sigma) at 37°C for 10 min and fixed in 10% buffered formalin. Images of the sections were acquired using a digital camera and the survival area was measured using ImageJ software. For each brain, the surviving area of brain tissue was calculated as the ratio of the area of ipsilateral TTC-stained tissue (non-ischemic) to the area of contralateral TTC-stained tissue.

### Sample preparation

At 3, 6, 12, 24, 48, 72 h, 7 days or 4 weeks after HI injury, the animals were deeply anesthetized with an overdose of sodium pentobarbital and then perfused transcardially with cold 0.9% saline, followed by 4% paraformaldehyde (PFA) in PBS. The brains were removed and post-fixed in PFA overnight, then cryoprotected with 30% sucrose for 48 h. Serial coronal sections (30 *µ*m) were cut and stored at −20°C.

### Cresyl violet staining

For cresyl violet staining, the sections obtained on day 7 were incubated with cresyl violet for 30 min at 37°C. Ethanol solution differentiated the stain. The sections were then rinsed with distilled water and fully air dried.

### Fluoro-Jade B (FJB) staining

FJB staining was performed as previously described (16). For FJB staining, the sections obtained at 3–72 h were incubated with a solution of 0.06% potassium permanganate for 30 min, and then incubated with a 0.0004% solution of FJB (AG310; Millipore, Billerica, MA, USA) and 4′,6-diamidino-2-phenylindole (DAPI; Sigma) for 20 min. The sections were then rinsed with distilled water and fully air dried. The number of FJB-positive neurons in 3 sections was determined in the ipsilateral hippocampus that was affected by HI injury.

### 3-Bromodeoxyuridine (BrdU) staining

For BrdU labeling, the rat pups were administered daily i.p. injections (50 *µ*g/g in 0.9% saline; Sigma) from day 1 to day 7 post-HI injury. BrdU^+^ cells were detected at 72 h (P10) or 4 weeks (P35) after surgery using an antibody against BrdU (OBT0030, rat; 1:400; Accurate Chemical & Scientific Corp., Westbury, NY, USA). Briefly, the brain sections were incubated with blocking solution (0.1% Triton X-100, 1% bovine serum albumin, and 5% normal goat serum in PBS) for 1 h at room temperature, followed by an overnight incubation with primary antibody (anti-rat BrdU; 1:400, OBT0030; Accurate Chemical & Scientific Corp.) at 4°C. The sections were then washed with PBS and incubated with a secondary antibody (Jackson ImmunoResearch Laboratories, Inc., West Grove, PA, USA) at room temperature for 1 h. After being treated with DAPI for 2 min, the sections were washed with PBS and mounted on slides with Fluoromount G (Cat. no. 17984-25; Electron Microscopy Sciences, Hatfield, PA, USA).

### Immunofluorescence staining

The differentiation states of the BrdU^+^ neurons were determined by co-labeling with an antibody against doublecortin (DCX) (AB2253, guinea pig; 1:1,500; Millipore) at 72 h following HI injury (P10) or with an antibody against neuronal nuclei antigen (NeuN) (MAB377, mouse; 1:100; Millipore) at 4 weeks following HI injury (P35).

Following immunostaining, the sections were analyzed by light microscopy at a magnification, ×20 using an inverted microscope (Zeiss Axiovert 200M; Carl Zeiss, Göttingen, Germany) interfaced with a digital camera (Zeiss Axion Cam MRc5; Carl Zeiss) controlled by a computer.

### Statistical analysis

Data are presented as the means ± standard error of the mean. Statistical differences between >2 groups were analyzed by using a one-way analysis of variance followed by the Turkey multiple comparison test. A value of P<0.05 was considered to indicate a statistically significant difference.

## Results

### Neuroprotective effects of the combination of EP and IGF-I in cultured neurons under conditions of OGD

**Figure 1:**
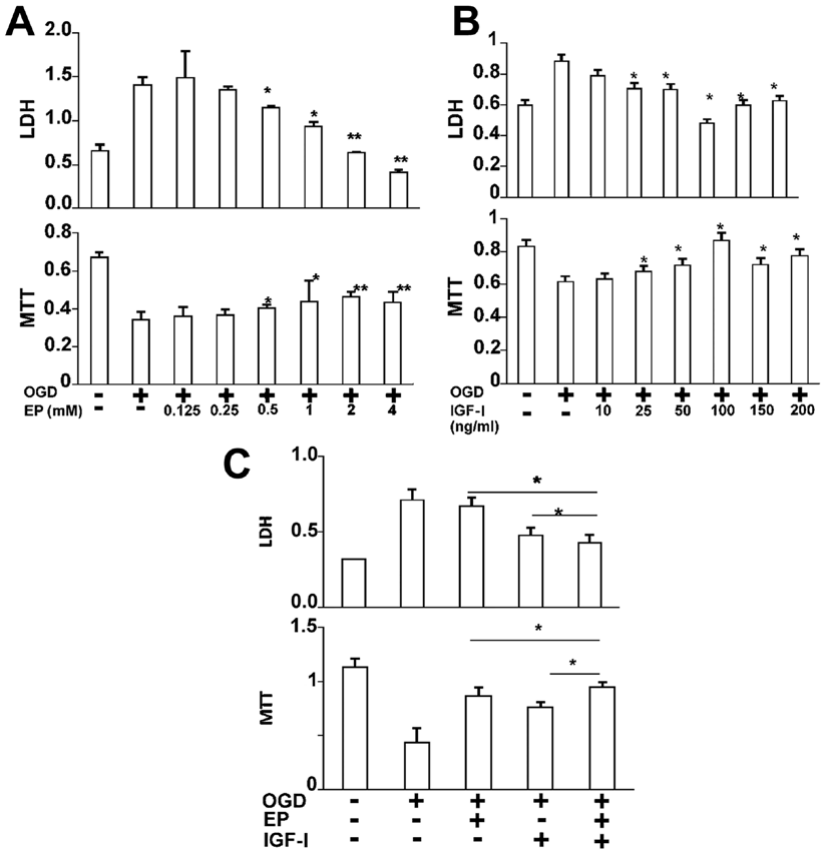
Ethyl pyruvate (EP) and insulin-like growth factor-I (IGF-I) protect primary cortical neurons against oxygen glucose deprivation (OGD). (A) EP reduced OGD-induced neuronal cell injury in a dose-dependent manner, as indicated by the reduced lactate dehydrogenase (LDH) levels in the culture medium and the increased MTT levels in the cell lysates at 24 h of reoxygenation. ^*^P<0.05 and ^**^P<0.01, compared with OGD alone (n=6). (B) IGF-I reduced OGD-induced neuronal injury in a dose-dependent manner, as indicated by the reduced LDH levels in the culture medium and the increased MTT levels in the cell lysates at 24 h of reoxygenation. ^*^P<0.05 compared with OGD alone (n=6). (C) Combined treatment with EP (0.5 mM) and IGF-I (25 ng/ml) further reduced OGD-induced neuronal cell injury than with each treatment alone. ^*^P<0.05 as indicated (n=6).

To examine the neuroprotective effects of EP and/or IGF-I against HI injury, we first examined the degree of neuronal injury by LDH assay and cell viability (by MTT assay) in the absence or presence of increasing concentrations of EP or IGF-I under conditions of OGD (17). Under conditions of OGD, the LDH levels were increased in the culture medium and the MTT levels were decreased in the cell lysate at 24 h of reoxygenation, suggesting neuronal injury under oxidative stress (Fig. 1A and B). As the concentration of EP or IGF-I increased in the culture medium, the LDH levels decreased and the MTT levels increased (Fig. 1A and B), both in a dose-dependent manner, indicating a decrease in neuronal injury and an increase in neuronal cell viability. The neuroprotective effects were the most prominent when EP (0.5 mM) was combined with IGF-I (25 ng/ml) as compared to treatment with EP or IGF-I alone (Fig. 1C).

### EP and IGF-I promote long-term behavioral development following HI injury

**Figure 2:**
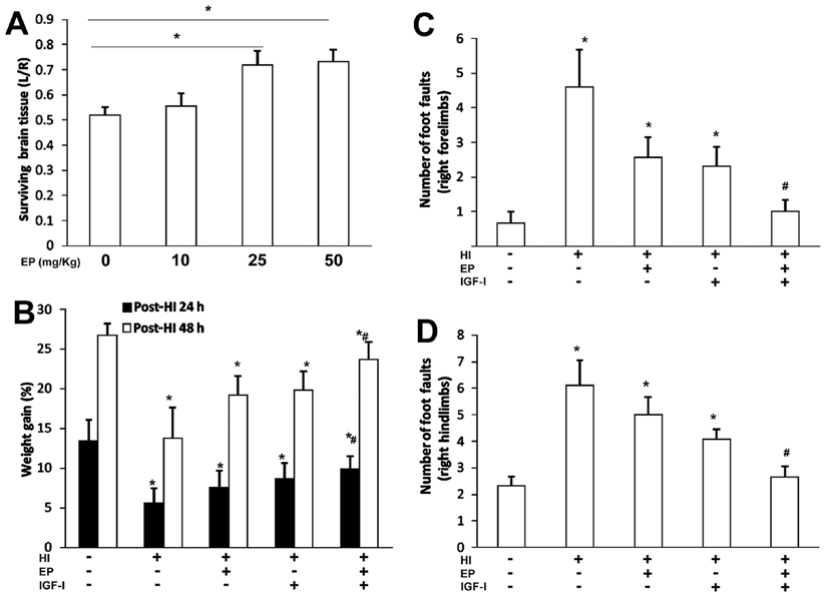
Ethyl pyruvate (EP) and insulin-like growth factor-I (IGF-I) promote long-term behavioral development following hypoxic-ischemic (HI) injury. (A) EP exerted neuroprotective effects in a dose-dependent manner (30 min after HI injury) ^*^P<0.05 as indicated (n=6). (B) Body weight following HI injury. Rat pups were treated with EP 30 min after HI injury or with IGF-I 24 h after HI injury or with both agents. The y-axis represents the percentage changes in body weight as compared to the weight before HI injury. ^*^P<0.05 compared with the sham-operated group, ^#^P<0.05 compared with other the 3 groups subjected to HI injury (n=8). Foot fault tests were performed at 4 weeks of recovery (P35). Number of foot faults of (C) right forelimbs or (D) right hindlimbs per 50 steps were counted within 5 min. ^*^P<0.05 compared with sham-operated group, ^#^P<0.05 compared with other 3 groups subjected to HI injury (n=8). L, left; R, right.

We then evaluated the neuroprotective effects of EP and/or IGF-I in a commonly used neonatal rat model of HI injury (18). At 48 h of recovery, EP began to show protective effects against HI injury at the dose of 25 mg/kg (administered at 30 min after HI injury), indicated by an increase in the volume area of surviving brain tissue (Fig. 2A). We selected a previously published IGF-I (3 mg/kg, 24 h post HI) treatment dose and schedule (19) to evaluate the neuroprotective effects of the two treatments.

Compared with the sham-operated rats, HI injury to the ipisilateral section of the brain resulted in weight loss within 24 h (Fig. 2B). While the rats in the sham-operated group gained approximately 13% of body weight the following day, the rats in the treated groups gained more weight over the same time period, with the combined treatment group gaining the most weight (Fig. 2B).

We employed a foot fault test to compare the sensorimotor behavior of the rats in the different treatment groups with that of the rats in the sham-operated group at 4 weeks of recovery (Fig. 2C and D). When the total number of foot faults per 50 steps was recorded within 5 min, the rats in the vehicle-treated group showed a significantly increased number of foot faults compared with the rats in the sham-operated group (right forelimb, Fig. 2C; right hindlimb, Fig. 2D). However, treatment with EP or IGF-I decreased the number of foot faults, and combination treatment with EP and IGF-I resulted in a further reduction in the number of both right forelimb and hindlimb foot faults compared with either treatment alone.

### Neuroprotective effects of the combination of EP and IGF-I in vivo

**Figure 3:**
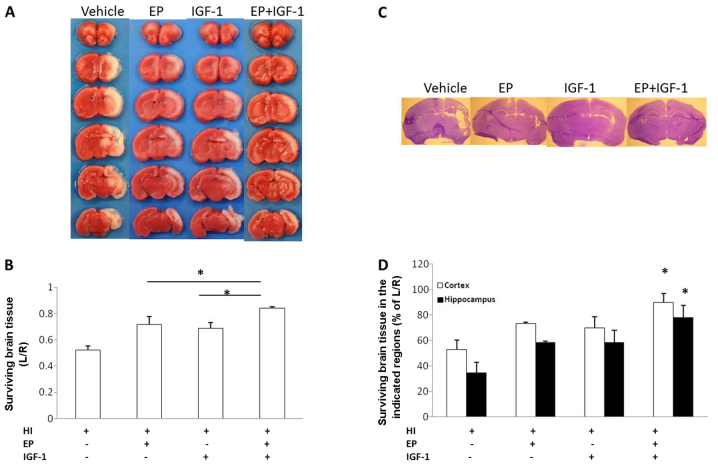
Ethyl pyruvate (EP) and insulin-like growth factor-I (IGF-I) reduce hypoxic-ischemic (HI) injury to neonatal rat brains. (A) Representative 2,3,5-triphenyltetrazolium chloride-stained brain sections from 9-day-old rats (48 h after HI injury), treated with the vehicle, EP (25 mg/kg, 30 min post HI), IGF-I (3 mg/kg, 24 h post-HI injury) or a combination of the EP and IGF-I. (B) Quantification of the amount of surviving brain tissue following the different treatments. ^*^P<0.05 as indicated (n=8). (C) Representative cresyl violet-stained sections from 14-day-old rats (7 days post-HI injury). (D) Quantification of the amount of surviving cortical or hippocampal tissue. ^*^P<0.05 compared with the other 3 groups subjected to HI injury (n=8). L, left; R, right.

At 48 h of recovery, the amount of surviving brain tissue in the EP + IGF-I group was 84.2±9.0%, significantly higher than that with EP treatment alone (71.8±14.2%) or IGF-I treatment alone (68.8±10.3%). These neuroprotective effects were additive and not transient (Fig. 3A and B). At 7 days of recovery, the amount of surviving brain cortex tissue in the EP + IGF-I group was 89.7±6.8%, significantly higher than that with EP treatment alone (73.2±1.4%) or IGF-I treatment alone (70.0±8.6%). The amount of surviving tissue in the hippocampus in the EP + IGF-I group was 78.0±9.6%, significantly higher than that with EP treatment alone (58.3±1.2%) or IGF-I treatment alone (58.5±9.3%) (Fig. 3C and D). Therefore, treatment with EP (25 mg/g, 30 min post-HI injury) in combination with IGF-I (3 mg/kg, 24 h post-HI injury) exerted neuroprotective effects against HI injury, as indicated by the increase in the volume of surviving brain tissue at 48 h (Fig. 3A and B) and 7 days (Fig. 3C and D) of recovery.

### Treatment with EP and IGF-I decreases apoptosis in the damaged hippocampus following HI injury

**Figure 4:**
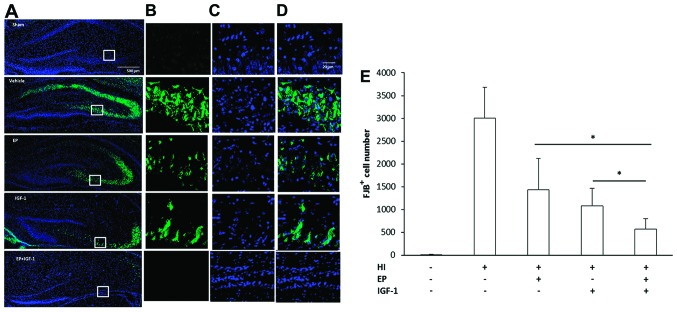
Combined treatment with ethyl pyruvate (EP) and insulin-like growth factor-I (IGF-I) decreases hypoxic-ischemic (HI) injury-induced neuronal cell death in the hippocampal cornu ammonis 3 (CA3) region. (A) At 72 h post-HI injury, the injured hippocampal neurons were identified by Fluoro-Jade B (FJB) staining (green) and the nucleus were stained with 4′,6-diamidino-2-phenylindole (DAPI) (blue). (B) High magnification view [squares in (A)] showing the morphology of degenerating hippocampal neurons (green). (C) Cell nuclei were identified by DAPI staining. (D) Merged image of (B) and (C). (E) Quantification of FJB^+^ cells in the ipsilateral hippocampus. ^*^P<0.05 as indicated (n=3–6 per group).

The increase in the volume of surviving tissue in the brain may have been a result of a reduced number of injured neurons. To clarify this, we labeled the injured neurons with FJB beginning at 3 h after injury and for up to at least 7 days. Fig. 4 illustrates the distribution of FJB^+^ cells in the hippocampus. The FJB^+^ neurons were detected at 3 h of recovery and reached peak numbers from 48–72 h (data not shown). At 72 h of recovery, HI injury to the neonatal brain increased the number of FJB^+^ cells both in the dentate gyrus (DG) and cornu ammonis 3 (CA3) region of the hippocampus (Fig. 4A–D). At a higher magnification, the FJB^+^ neurons displayed distinct apoptotic nuclei with either a condensed or fragmented morphology. Compared with the vehicle-treated group, the numbers of FJB^+^ neurons were significantly decreased by EP treatment or IGF-I treatment, whereas combined treatment with EP and IGF-I led to a further decrease in the number of FJB^+^ neurons compared to the groups treated with EP or IGF-I alone (Fig. 4E).

### HI injury promotes neuronal cell proliferation

**Figure 5:**
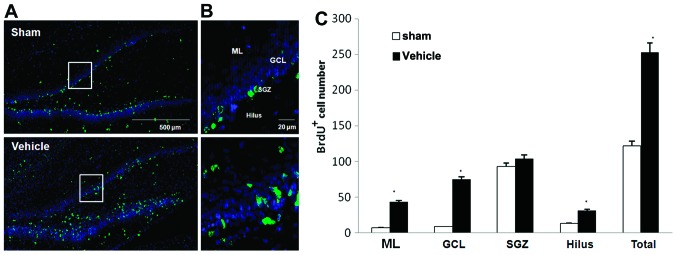
Hypoxic-ischemic (HI) injury promotes neuronal cell proliferation. (A) HI injury promoted cell proliferation in the hippocampus. Proliferating cells were identified by bromodeoxyuridine (BrdU) immunostaining (green) 72 h post-HI injury. The structure of the dentate nucleus is shown by the alignment of nuclei stained with 4′,6-diamidino-2-phenylindole (blue). (B) High magnification view of the square in (A) showing the distribution of proliferating cells. (C) Quantification of proliferating cells in the different subregions of the hippocampus. ^*^P<0.05 compared with sham-operated group (sham; n=6). ML, molecular layer; GCL, granule cell layer; SGZ, subgranular zone.

To determine the effect of HI injury on neurogenesis, we labeled proliferating cells with BrdU during the first 7 days following HI injury. Within the hippocampus of the rats in the sham-operated group, the majority of BrdU^+^ cells was distributed in the subgranular zone (SGZ), where neural stem cells or immature neurons reside (Fig. 5). In comparison, the BrdU^+^ cells in the rats in the group subjected to HI injury were not confined to the SGZ, but were scattered around the entire DG, suggesting that newborn cells had migrated to other locations. Overall, the number of BrdU^+^ cells in the DG of the rats in the group subjected to HI injury was double that of the cells in the DG of rats in the sham-operated group (Fig. 5C).

### Treatment with EP and IGF-I promotes neurogenesis in the damaged hippocampus following HI injury

**Figure 6:**
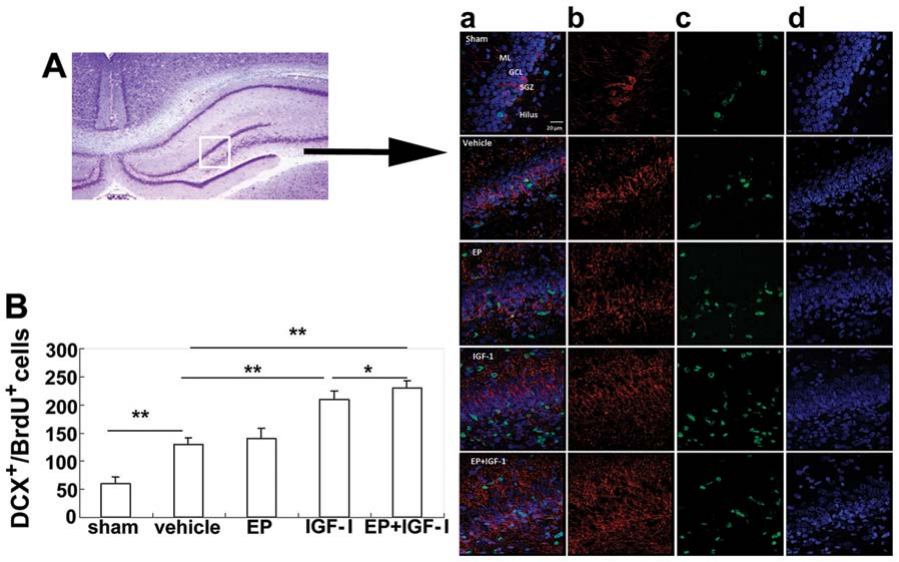
Combined treatment with insulin-like growth factor-I (IGF-I) and ethyl pyruvate (EP) increases hypoxic-ischemic (HI) injury-induced neurogenesis. (A) High magnification view of the square in the hippocampus. Double immunostaining to identify the newborn neurons (panel a, merge) with doublecortin (DCX) immunostaining (panel b, red) and bromodeoxyuridine (BrdU) immunostaining (panel c, green) 72 h post-HI injury. Nuclei are stained with DAPI (panel d, blue). (B) Quantification of the number of newborn neurons (DCX^+^BrdU^+^). ^*^P<0.05, ^**^P<0.01 as indicated (n=6).

**Figure 7:**
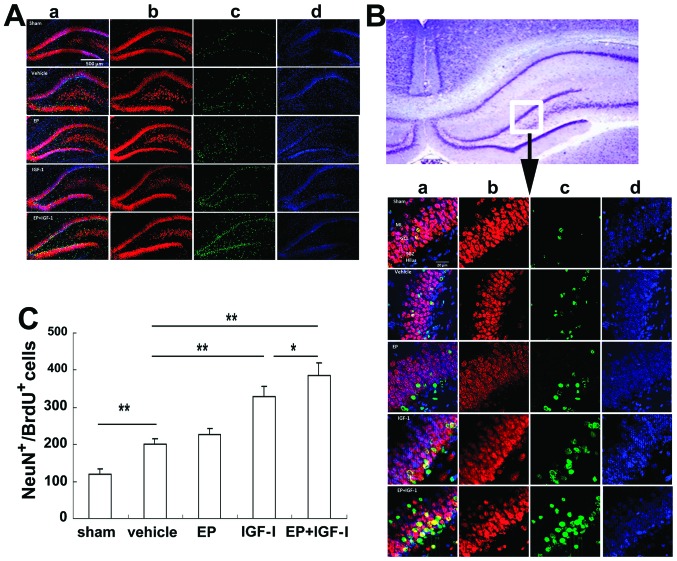
Combined treatment with insulin-like growth factor-I (IGF-I) and ethyl pyruvate (EP) promotes the maturation of newborn neurons. (A) Matured neurons born in the hippocampus. Double immunostaining to identify the matured neurons (panel a, merge) with neuronal nuclei antigen (NeuN) (panel b, red) and bro-modeoxyuridine (BrdU) (panel c, green) at 4 weeks post-hypoxic-ischemic (HI) injury. Nuclei are stained with DAPI (blue; panel d). (B) High magnification view of the square in the hippocampus to show the mature neurons (panel a, merge) with NeuN antibody (panel b) and antibody against BrdU (panel c) 4 weeks post-HI injury. Nuclei are stained with DAPI (blue;panel d). (C) Quantification of the number of newborn neurons (NeuN^+^BrdU^+^). ^*^P<0.05, ^**^P<0.01 as indicated (n=6).

To examine the neuronal differentiation of BrdU^+^ cells, we double labeled BrdU^+^ cells with either DCX, a marker for immature neurons, or NeuN, a marker for mature neurons. At 72 h post-HI injury, the number of BrdU^+^DCX^+^ cells in the group subjected to HI injury was increased compared to that of the rats in the sham-operated group (Fig. 6). Although mainly distributed in the SGZ of the rats in the sham-operated group, the BrdU^+^DCX^+^ cells were also distributed in the granule cell layer (GCL) in the group subjected to HI (Fig. 6A, panel d), indicating that more BrdU^+^DCX^+^ cells may have migrated. Although the numbers of the BrdU^+^DCX^+^ cells in the group treated with EP were similar to those of the vehicle-treated group, the numbers were markedly increased in the IGF-I-treated group and the combined treatment group, indicating that IGF-I, but not EP, stimulated neurogenesis (Fig. 6B). At 4 weeks post-injury, the number of NeuN^+^BrdU^+^ neurons was also elevated (Fig. 7) in the group subjected to HI compared to the sham-operated group. Of note, the increase in the number of DCX^+^ cells (Fig. 6B) and NeuN^+^ cells (Fig. 7C), showing a similar pattern among the treatment groups, suggesting that IGF-I not only stimulated neurogenesis, but also neuronal survival and differentiation.

## Discussion

HI injury results in severe damage to immature brains as it disturbs multiple developmental events in addition to normal brain functions. Compared with the adult brain, neonatal brains often display different forms of cell death (20) and different responses to therapeutic interventions (21,22). One of the underlying causes for these differences is the inability of the immature brains to handle the rapidly accumulating levels of oxidative stress immediately following HI injury (1–3). In fact, treatments with antioxidants provide beneficial effects as has been shown in animal models of HI injury (14,23,24). While the removal of oxidants only offers temporary relief, resumption of the normal developmental process is equally urgent for the young brain. In contination of our previous study on the beneficial effects of SP (14), in this study, we focused on the neuroprotective effects of EP and IGF-I.

Our choice of this combination was based on the following considerations: first, SP provided long-term neuroprotective effects to immature brains following HI, but it is not suitable for clinical use due to its short half-life, instability and side-effects in adults (25). It has been shown that EP, a more stable and lipophilic derivative of pyruvic acid (25), exerts neuroprotective effects on cortical neurons equal to those of SP under conditions of OGD. More importantly, EP (25 mg/kg) reduced brain injury to the same extent (~20%) as SP (500 mg/kg), but at a much lower dose (data not shown). Due to its anti-inflammatory effects (26), EP is evidently the better choice for further investigation. These positive results showing the neuroprotective effects of EP, are contrast to those of another study demonstrating the negative effects of neuroprotectants (27). The results in that case showed no reduction in the severity of infarction in an *in vivo* rat model, despite the fact that the model used was similar to the one we used. The main differences between the studies were that the rats were a different strain (Wistar compared to Sprague-Dawley), the doses were lower than our doses (10 and 40 mg/kg compared to our most effective doses at 25 and 50 mg/kg) and the exposure to hypoxia was shorter (50 min compared to 150 min). The outcome was scored only in terms of macroscopic brain injury so there is a possibility that some effects were missed. The exact reasons for these different results warrant further investigation, as it is important that these effects be reproducible in different model systems if their potential for clinical treatment is to be fulfilled (27).

Second, IGF-I is a pleiotrophic factor essential to the survival of immature neurons and maintenance of the high metabolism needed to fulfill developmental needs, such as neuronal migration, axon extension and synaptogenesis (28). Serum IGF-I levels have been shown to be decreased in human newborns suffering from HIE (29) and neuronal IGF-I mRNA (30) and IGF-I serum levels have been shown to be decreased in neonatal rat brains following HI injury (19,31). However, immediate intraventricular (32) and intranasal delivery (33) was less effective than delayed subcutaneous delivery (24 and 48 h) in terms of the reduction of the voluem of injured tissue and long-term behavioral recovery. This improvement with delayed treatment indicates that IGF-I is less effective during the acute phase when oxidative stress is at its highest. A similar result was observed in a study on rat oligodendrocyte progenitors, where delayed cell death caused by glutamate was prevented by both the immediate and late (16 h post-exposure) administration of IGF-I, while cell proliferation was promoted (34). In addition IGF-I reverse the loss in oligodendrocyte transcription factor-positive cells in white matter following HI injury (34). Therefore, combining IGF-I with early EP treatment to reduce oxidative stress may protect immature neurons against injury and assist their normal development.

The above hypothesis was supported by the results of the present study: early EP treatment combined with the delayed s.c. injection of IGF-I led to reduced neuronal cell death under conditions of OGD, and reduced damage to the neonatal brain due to HI injury, both in short-term and long-term experiments. In the developing hippocampus, the DG is known to be more vulnerable to HI injury than the CA3 region (36). In this study, we found that neuronal cell death occurred both in the DG and CA3 regions, which may result from a longer exposure to hypoxia (2.5 h) than in other studies (usually <2 h) (35–37). At 72 h of recovery, treatment with EP and IGF-I alone reduced the number of injured neurons, while combined treatment further reduced the number of FJB^+^ neurons. These neuroprotective effects may be a result of their complimentary neuroprotective actions. IGF-I promotes neuronal cell survival by activating the key survival signaling kinase, Akt (38), whereas EP scavenges free radicals and reduces inflammatory reactions (15).

Apart from promoting neuronal cell survival, IGF-I also stimulates neurogenesis (39,40). Unlike adult brains, to which traumatic brain injury mainly induces the proliferation of reactive astrocytes (41), HI injury to neonatal mouse brains promotes the proliferation of multiple cell types, including microglia, endothelial cells, oligodendrocytes and neurons (42). Following HI injury, we found that the BrdU^+^ cells were mainly distributed in the granular and molecular neuronal cell layers of the hippocampus, which contain neurons from secondary neurogenesis (43). To characterize neurogenesis and differentiation following HI injury, we double stained BrdU^+^ cells with DCX, a marker for immature neurons, or NeuN, a marker for mature neurons. At 3 days and 4 weeks following HI injury, a parallel trend emerged: while EP alone did not have much of an effect, IGF-I or combined treatment with both EP and IGF-I resulted in a similar increase in the number of BrdU^+^DCX^+^ immature neurons, as well as in the number of BrdU^+^NeuN^+^ mature neurons. In addition, a greater number of BrdU^+^DCX^+^ cells had migrated into the GCL and had likely become incorporated into the neuronal network, as demonstrated by improved motor coordination at 4 weeks of recovery.

In conclusion, the findings of this study demonstrate that combining early EP administration with the delayed s.c. injection of IGF-I exerts neuroprotective effects on immature neurons under conditions of OGD and following HI injury. These effects were likely the result of a reduction in neuronal cell death and an increase in neurogenesis/differentiation following HI injury.
